# Development of Modified Drug Delivery Systems with Metformin Loaded in Mesoporous Silica Matrices: Experimental and Theoretical Designs

**DOI:** 10.3390/pharmaceutics17070882

**Published:** 2025-07-04

**Authors:** Mousa Sha’at, Maria Ignat, Florica Doroftei, Vlad Ghizdovat, Maricel Agop, Alexandra Barsan (Bujor), Monica Stamate Cretan, Fawzia Sha’at, Ramona-Daniela Pavaloiu, Adrian Florin Spac, Lacramioara Ochiuz, Carmen Nicoleta Filip, Ovidiu Popa

**Affiliations:** 1 Department of Pharmaceutical Technology, Faculty of Pharmacy, “Grigore T. Popa” University of Medicine and Pharmacy, 16 Universității Street, 700115 Iasi, Romania; mousa-shaat@umfiasi.ro (M.S.); alexandra.m.bujor@umfiasi.ro (A.B.); monica.stamate@umfiasi.ro (M.S.C.); lacramioara.ochiuz@umfiasi.ro (L.O.); 2 Laboratory of Material Chemistry, Department of Chemistry, “Alexandru Ioan Cuza” University of Iasi, Bv. Carol I, No. 11, 700506 Iasi, Romania; 3 Department of Inorganic Polymers, Petru Poni Institute of Macromolecular Chemistry, 41A Grigore Ghica Voda Alley, 700487 Iasi, Romania; florica.doroftei@icmpp.ro; 4 Biophysics and Medical Physics Department, “Grigore T. Popa” University of Medicine and Pharmacy, 700050 Iasi, Romania; vlad.ghizdovat@umfiasi.ro; 5 Department of Physics, “Gheorghe Asachi” Technical University of Iasi, 700050 Iasi, Romania; magop@tuiasi.ro; 6 Academy of Romanian Scientists, 3 Ilfov, 050044 Bucharest, Romania; 7 National Institute for Chemical-Pharmaceutical Research and Development—ICCF, 112 Vitan Avenue, 031299 Bucharest, Romania; fawzya.shaat@gmail.com (F.S.); pavaloiu_daniella@yahoo.com (R.-D.P.); 8 Department of Physico—Chemistry, Faculty of Pharmacy, “Grigore T. Popa” University of Medicine and Pharmacy, 16 Universității Street, 700115 Iasi, Romania; adrian.spac@umfiasi.ro; 9 Department of Neurology, “Grigore T. Popa” University of Medicine and Pharmacy, 700115 Iasi, Romania; carmen.filip@umfiasi.ro; 10 Department of Neurology 2, “Prof. Dr. N. Oblu” Emergency Clinical Hospital, 700309 Iasi, Romania; 11 Department of Emergency Medicine, “Grigore T. Popa” University of Medicine and Pharmacy, 700115 Iasi, Romania; ovidiu.popa@umfiasi.ro

**Keywords:** KIT-6, metformin hydrochloride, mathematical models, mesoporous silica matrix, dissolution test

## Abstract

**Background/Objectives:** Mesoporous silica materials, particularly KIT-6, offer promising features, such as large surface area, tunable pore structures, and biocompatibility, making them ideal candidates for advanced drug delivery systems. The aims of this study were to develop and evaluate an innovative modified-release platform for metformin hydrochloride (MTF), using KIT-6 mesoporous silica as a matrix, to enhance oral antidiabetic therapy. **Methods:** KIT-6 was synthesized using an ultrasound-assisted sol-gel method and subsequently loaded with MTF via adsorption from alkaline aqueous solutions at two concentrations (1 and 3 mg/mL). The structural and morphological characteristics of the matrices—before and after drug loading—were assessed using SEM-EDX, TEM, and nitrogen adsorption–desorption isotherms (the BET method). In vitro drug release profiles were recorded in simulated gastric and intestinal fluids over 12 h. Kinetic modeling was performed using seven classical models, and a multifractal theoretical framework was used to further interpret the complex release behavior. **Results:** The loading efficiency increased with increasing drug concentration but nonlinearly, reaching 56.43 mg/g for 1 mg/mL and 131.69 mg/g for 3 mg/mL. BET analysis confirmed significant reductions in the surface area and pore volume upon MTF incorporation. In vitro dissolution showed a biphasic release: a fast initial phase in an acidic medium followed by sustained release at a neutral pH. The Korsmeyer–Peppas and Weibull models best described the release profiles, indicating a predominantly diffusion-controlled mechanism. The multifractal model supported the experimental findings, capturing nonlinear dynamics, memory effects, and soliton-like transport behavior across resolution scales. **Conclusions:** The study confirms the potential of KIT-6 as a reliable and efficient carrier for the modified oral delivery of metformin. The combination of experimental and multifractal modeling provides a deeper understanding of drug release mechanisms in mesoporous systems and offers a predictive tool for future drug delivery design. This integrated approach can be extended to other active pharmaceutical ingredients with complex release requirements.

## 1. Introduction

Nanomedicine, at the global scale, primarily focuses on the application of nanotechnology in drug formulation, utilizing both organic and inorganic nanomaterials, which are already integrated into the pharmaceutical industry. Advanced drug delivery systems enable the efficient transport of active substances to specific cells or tissues, facilitating targeted and controlled release. Examples of such systems include biodegradable polymers, hydroxyapatite, xerogels, hydrogels, and mesoporous silica [1,2,3,4].

Mesoporous silica nanoparticles (MSNs) represent a promising class of nanomaterials widely employed in the biomedical field, particularly in modern drug delivery systems. They stand out due to their well-defined porous architecture, large specific surface area, and easy surface functionalization, making them versatile carriers for the transport and controlled release of various bioactive compounds [5,6]. Their porous structure enables the efficient loading of therapeutic molecules of different sizes—from conventional drugs to proteins, nucleic acids, or antigens—thus, improving their solubility and bioavailability [7,8,9].

A major advantage of MSNs lies in their ability to achieve targeted and stimuli-responsive drug release. These systems can respond to specific biological or physicochemical triggers, such as pH, enzymes, temperature, light, or magnetic fields. This allows for the controlled and localized release of the active substance, reducing side effects and enhancing the therapeutic efficacy. Furthermore, surface functionalization improves the biocompatibility of MSNs and reduces their potential toxicity [10,11,12,13].

Mesoporous silica materials, particularly KIT-6, have emerged as promising candidates for advanced drug delivery systems (DDSs) due to their unique structural properties and functionalization potential. KIT-6 is a type of mesoporous silica with a well-defined three-dimensional cubic structure that provides a large surface area, tunable pore size, and excellent stability [14]. These attributes enable KIT-6 to effectively encapsulate and release a wide range of therapeutic agents, positioning it as a key material in the field of nanomedicine.

KIT-6 has been investigated for the delivery of various drugs, including anticancer agents, anti-inflammatory compounds, and antimicrobial agents [15,16,17,18,19]. By adjusting the design parameters of the delivery systems, researchers have enhanced drug bioavailability and therapeutic efficacy while reducing associated side effects. Another advantage of using KIT-6 lies in the potential for the modified release of active substances, which can be achieved through multiple mechanisms: diffusion (where drug molecules passively exit the pores), stimuli-responsive release (where functionalization enables release in response to specific external stimuli, such as temperature, pH, or irradiation), and desorption-controlled release (where the desorption rate can be fine-tuned for gradual drug delivery) [20,21].

The active pharmaceutical ingredient, metformin hydrochloride (1,1-dimethylbiguanide, or MTF, C_4_H_12_ClN_5_), is an antidiabetic agent widely used as a first-line therapy in type-2 diabetes mellitus.

Since the early 2000s, nanotechnology-based therapies utilizing mesoporous silica nanoparticles (MSNs) have been increasingly developed for the management of diabetes mellitus. These systems aim to enhance biopharmaceutical efficacy and address challenges associated with conventional chronic treatments, particularly the maintenance of stable blood glucose levels throughout the day [22].

In this study, the main objective was to develop a controlled release system for metformin hydrochloride using a mesoporous silica matrix (KIT-6). To achieve this, the synthesis of the silica matrix, the drug-loading process, the physicochemical characterization of both the loaded and unloaded matrices, textural analysis, determination of the loading efficiency, and evaluation of the release kinetics through in vitro dissolution tests were conducted. Our research aims to provide a comprehensive perspective on the application of mesoporous silica matrices in pharmaceutical formulations, thereby contributing to the development of efficient and innovative drug delivery systems.

## 2. Materials and Methods

### 2.1. Chemicals and Reagents

The mesoporous silica matrices were synthesized using the following reagents: Pluronic PEO_20_-PPO_70_-PEO_20_ (or P_123_ with Mr = 5800), which was obtained from Sigma Aldrich Chemie GmbH, Taufkirchen, Germany; TEOS (98% pure), which was purchased from Merck (Darmstadt, Germany); and hydrochloric acid (HCl, ≥37.0%, denoted as conc. HCl) and n-butanol (C_4_H_10_O; Mr = 74.12), which were obtained from Chemical Company S.A. (Iasi, Romania). The principal active substance used in the study was metformin hydrochloride (or MTF) (97% pure), which was obtained from Sigma Aldrich Chemie GmbH (Steinheim, Germany). The following mobile-phase reagents were required for the quantitative determination of the MTF in the samples: sodium acetate (≥99.0% pure)–Silal Trading SRL (Bucharest, Romania); glacial acetic acid (99.9% pure), which was obtained from Chimreactiv SRL (Bucharest, Romania); and methanol Chromasolv™ for HPLC (≥99.9% pure), which was supplied from Honeywell Riedel-de Haën (Seelze, Germany). In vitro dissolution studies required dissolution media obtained using potassium dihydrogen phosphate (≥99.5% pure)–Utchim SRL (Ramnicu Valcea, Romania)–and glacial acetic acid (99.9% pure)–Chimreactiv SRL (Bucharest, Romania), and from Chemical Company S.A. (Iasi, Romania), we purchased the following reagents: sodium hydroxide (98.5% pure) and potassium chloride (≥99.0% pure). To prepare the deionized water needed for the experiments, an ELGA Purelab Ultra water system was used. A GFL-type 2004 laboratory equipment no. 11918315J distiller (Burgwedel, Germany) was used to obtain distilled water. The reagents used did not require any additional purification.

### 2.2. Equipment

An Agilent Technologies 1200 (Santa Clara, CA, USA) liquid chromatograph; a Thermo Scientific ODS Hypersyl^TM^ column (250 × 4.6 mm, 5 μm), Vilnius, Lithuania; a microliter™ syringe, 20 μL, Hamilton Bonaduz AG (Bonaduz, Switzerland); and Agilent ChemStation 32 software (Rev. B.03.02.) were used. A pH meter (inoLAB pH 7110, Xylem Analytics Germany GmbH, Weilheim, Germany), a PH CHECK G 5040-0302 instrument (Dostmann GmbH, Wertheim, Germany), an analytical balance (PIONEER^®^ Analytical OHAUS PX124M, Ohaus Corporation, Parsippany, NJ, USA), a water bath (Biobase model SY-1L4H, Biobase (Shandong) Co., Ltd., Jinan, China), an ultrasonic bath (Biobase model UC-40A, Biobase (Shandong) Co., Ltd., Jinan, China), a dissolution apparatus (SR 8 Plus Dissolution Test Station, model 73-100-104, Hanson Research, Chatsworth, CA, USA), a micropipette (Pipet4u^®^ Pro, 20–200 μL, article OK99957, AHN^®^ Biotechnologie GmbH, Nordhausen, Germany), a single-channel pipette (Transferpette^®^, Dig. 100–1000 μL, Brand GMBH + CO KG, Wertheim, Germany), and a single-channel microliter pipette (Rotilabo^®^–Mikroliterpipette, 0.5–5.0 mL, Carl Roth GmbH, Karlsruhe, Germany) were used. To obtain the mesoporous silica matrices, the following equipment was used: an ultrasonic probe (model CV33)—VIBRA CELLTM AMPLITUDE (Sonics, Newtown, CT, USA), a centrifuge—ROTOFIX 32 A (Hettich Instruments, Beverly, MA, USA), a magnetic stirrer (VELP Scientifica, Usmate Velate, Italy), a LabTech LDO-060E oven (Daihan Labtech CO., Batam, Indonesia), and a calcination furnace—Carbolite^®^ Elf 11/14B (Derbyshire, UK).

### 2.3. KIT-6 Mesoporous Silica Synthesis

First, 9 g of the copolymer—P123—was weighed on an analytical balance and placed in a 1000 mL Berzelius beaker. Then, 325 mL of deionized water and 17.4 mL of HCl (37%, measured volumetrically with a graduated cylinder) were added, and the mixture was dissolved by magnetic stirring for 1 h at 40 °C. Then, 9 mL of n-butanol was added to the solution obtained. As soon as the TEOS was added dropwise, ultrasonication was started (amplitude: 25%; operation: 3 on/1 off cycles) and continued for 1 h (60 min), with gentle agitation every 5 to 10 min. The obtained silica matrix was separated from the supernatant by centrifugation at 4000 rpm for 10 min, and at the end, the matrix-washing step was carried out using deionized water, ethyl alcohol, and a water–alcohol mixture (1:1) for a minimum of 5–7 washing cycles by matrix entrainment. The washed mesoporous silica was left in an oven to dry (at 60 °C for 48–72 h). After drying, the material was subjected to calcination at 550 °C (at 1 °C/min) for 8 h in order to remove the organic compounds, leaving behind voids through the silica matrix to form the mesoporous structure.

### 2.4. Metformin Immobilization in the Mesoporous Silica Matrix

The loading of the KIT-6 matrix was performed by the adsorption technique from aqueous solutions (at pH 12) of metformin hydrochloride at concentrations of 1 and 3 mg/mL. The detailed method of the KIT-6 matrix loading was performed in the same manner as that of MCM-48, as described in a previously published study [22].

### 2.5. Quantitative Determination of the Metformin Concentration

The difference between the amount of MTF in the initial (loading) solution and the amount of MTF quantitatively determined from the filtered supernatant obtained after contact with the matrix was used to determine the degree of the loading, which is the ratio of the active substance to one gram of the silica matrix. An in-house developed, optimized, and verified procedure was used to inject samples into the HPLC instrument for the quantitative determinations [23].

### 2.6. SEM and EDX-SEM Analyses

A Quanta 200 scanning electron microscope (FEI Company, Hillsboro, OR, USA) was used to perform SEM-EDX experiments. Scanning electron microscopy (SEM) was used to determine the morphology of the unloaded KIT-6 matrix, as well as those of samples S1 and S2.

### 2.7. TEM Analysis

An HT7700 HITACHI microscope (Hitachi High-Technologies Corporation, Tokyo, Japan), running in high-contrast mode at a 100 kV acceleration voltage, was used for Transmission Electron Microscopy (TEM) research. Solid silica matrix particles were directly deposited onto a 300-mesh carbon-coated copper grid (Ted Pella, Redding, CA, USA) to prepare the samples.

### 2.8. Nitrogen Sorption Isotherms

The nitrogen adsorption–desorption method was used to determine the texture. The BET method’s specific surface area computations are based on measuring the quantity of nitrogen that has been adsorbed on—or desorbed from—solid surfaces. In order to evaluate the unloaded sample (KIT-6) and the metformin-loaded samples (S1 and S2), the N_2_ sorption isotherms were recorded using a Quantachrome Nova 2200 Instrument and a pore size–surface area analyzer (Quantachrome Instruments, Odelzhausen, Germany).

Procedure: The sample to be analyzed (~50 mg) is weighed into a quartz cell fitted with a tightly closing stopper, after which the sample is vacuum degassed (the moisture and any adsorbed gases are removed from the sample to be analyzed). After degassing, the cell containing the test sample is heated while exposing it to the action of an advanced vacuum, followed by cooling the sample to atmospheric pressure by filling the cell with a gas that does not adsorb on the test sample at room temperature, such as nitrogen. The test sample is again subjected to the action of the high-pressure vacuum, and the sample temperature is lowered to the temperature of liquid nitrogen (−196 °C = 77 K), thus promoting adsorption. The adsorbing gas is introduced in small portions to the tube containing the sample to be analyzed, and the pressure evolution is monitored.

An adsorption isotherm is a graphical representation of the amount of gas adsorbed by the sample as a function of the gas pressure at a constant temperature. Following that, the data and information are processed in accordance with adsorption theories, and the Brunauer–Emmett–Teller method is taken into consideration for calculating the BET specific surface area in square meters per gram [24]. The volume of the adsorbed liquid nitrogen is measured to determine the overall pore volume. The Barrett–Joyner–Halenda (BJH) model and the desorption portion of the isotherm are used to estimate the average pore diameter.

### 2.9. In Vitro Dissolution Studies

The in vitro dissolution test for the MTF-loaded silica mesoporous matrices was performed in two simulated biological fluids with different pH values, namely, simulated gastric fluid (SGF) at pH 1.2 and simulated intestinal fluid (SIF) at pH 6.8, where the release profiles of the active molecule from the developed particulate systems were observed. The pre-set working conditions were as follows: apparatus no. 2 (with paddles), 50 mL of the dissolution medium (being SGF in the first 2 h and SIF in the following 12 h), a constant temperature of 37 ± 0.5 °C, stirring at 50 rpm, and sampling 1 mL of the sample in the first 0.5 h and then every hour until the end of the test. All the collected samples were filtered, and the quantitative determination of the MTF was performed by injecting the samples into an HPLC instrument according to an analytical method developed and validated in our laboratory [23]. The equipment used was an SR 8 Plus Series (AB & L Jasco, Cluj-Napoca, Romania).

### 2.10. Analysis of In Vitro Drug Release Kinetics

The analysis of the release kinetics of the MTF from KIT-6 was conducted by fitting the in vitro dissolution data to seven mathematical models: zero-order, first-order, Higuchi, Korsmeyer–Peppas, Weibull, Hixson–Crowell, and Baker–Lonsdale. The release profiles were analyzed using KinetDS version 3.0 software [25], with each model being assessed for its goodness-of-fit using the correlation coefficient (R^2^), root-mean-square error (RMSE), and Akaike Information Criterion (AIC). A reliable predictive model was considered to have an R^2^ value of close to 1 along with low RMSE and AIC values.

The data were expressed as means ± standard deviations, and the statistical significance was set at *p* < 0.05. Statistical comparisons between formulations S1 and S2 were performed using Student’s *t*-test and one-way ANOVA in LibreOffice (version 25.2.4). The 95% confidence intervals for the model parameters were also calculated using KinetDS version 3.0 software.

The following mathematical equations were used:

Zero-order kinetics:

(1) $$M_{t}=K_{0}\cdot t$$

First-order kinetics:

(2) $$M_{t}=100\cdot \left(1-e^{-kt}\right)$$

Higuchi model:

(3) $$M_{t}=K_{H}\cdot t^{0.5}$$

Korsmeyer–Peppas model:

(4) $$M_{t}=K_{p}\cdot t^{n}$$

Weibull model:

(5) $$M_{t}=1-exp\left[\frac{{-(t)}^{b}}{a}\right]$$

Hixson–Cromwell model:

(6) $$M_{t}^{\frac{1}{3}}=K_{HC}\cdot t+M_{0}^{\frac{1}{3}}$$

Baker–Lonsdale model:

(7) $$\frac{3}{2}\times \left[1-{\left(1-M_{t}\right)}^{2/3}\right]-M_{t}=K_{BL}\times t$$

where $M_{t}$ is the amount of the drug substance released at time *t*, $K_{0}$ is the zeroth-order rate constant of the drug release rate, *k* is the first-order yield rate constant, $K_{H}$ is the yield rate constant of the Higuchi model (Higuchi release constant), $K_{p}$ is the Korsmeyer–Peppas release rate constant, *n* is an exponential factor (an indicator of the drug release mechanism), *a* and *b* are Weibull parameters, *K_HC_* is the Hixson–Crowell release rate constant, *K_BL_* is the Baker–Lonsdale release rate constant.

## 3. Results and Discussion

### 3.1. Metformin-Loading Capacity

Following the chemical synthesis of KIT-6 by the ultrasound method, MTF was loaded by adsorption from aqueous solutions at pH 12 and concentrations of 1 and 3 mg/mL. In the case of sample S1, loaded with the solution at a concentration of 1 mg/mL, we have a loading degree of 56. 432 mg/g silica, in contrast to sample S2, where the use of a three-times higher concentration does not lead to a tripling of the degree of the loading, the value obtained being 131.686 mg/g silica (an increase of 233%) (Table 1). As observed, the reduced loading yield (73.16% vs. 94.05%), even though a higher concentration of the loading solution was used (131.69 mg/g vs. 56.43 mg/g), is most probably due to the matrix saturation with MTF molecules at the adsorption sites.

### 3.2. Scanning Electron Microscopy (SEM) Coupled with Energy-Dispersive X-Ray Spectroscopy (EDX-SEM)

**Figure 1:**
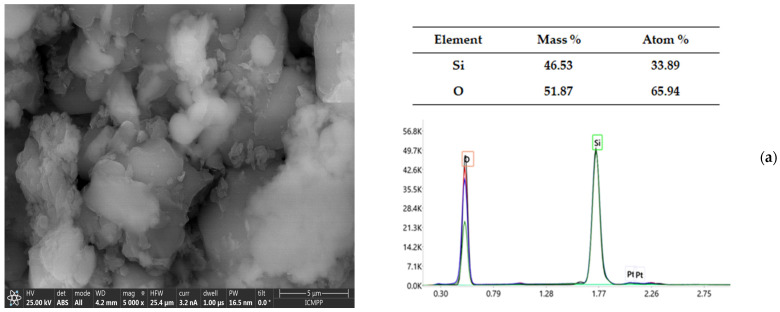

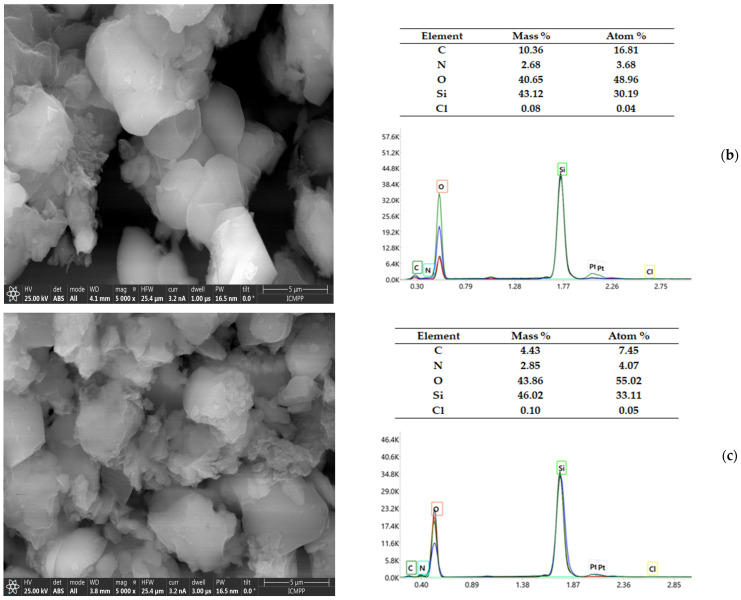
SEM images (**left**) and EDX spectra (**right**) of KIT-6 sample unloaded (**a**), KIT-6 sample S1 (**b**), and KIT-6 sample S2 (**c**).

The loaded (S1 and S2) and unloaded (KIT-6) silica matrices were analyzed using scanning electron microscopy (SEM) coupled with energy-dispersive X-ray spectroscopy EDX so that the experimental values obtained represent the average (the values in the tables included in Figure 1) of three different regions analyzed from each sample (EDX spectra in Figure 1). The compositional analysis for the unloaded matrices (KIT-6) reveals an average weight percentage and an average atomic percentage of silicon (Si) of 46.53% and 33.89%, respectively; for oxygen (O), the average weight percentage was 51.87%, and the average atomic percentage was 65.94%. These results confirm that the KIT-6-type matrix has a predominantly silica composition, with a Si/O ratio typical for a mesoporous silicon dioxide (SiO_2_) structure.

For sample S1 (KIT-6, 1 mg/mL, pH 12), the significant increases in the carbon (10.36% and 16.81%, respectively) and nitrogen (2.68% and 3.68%, respectively) contents indicate efficient MTF loading. The decreases in the percentages of Si (43.12% versus 30.19%) and O (40.65% versus 48.96%), compared to the Si and O percentages in the unloaded matrices, suggest that MTF occupies a part of the surface and pores in the mesoporous structure. As for the analysis of sample S2 (KIT-6, 3 mg/mL, pH 12), the higher loading concentration maintains the trends observed at the 1 mg/mL concentration but with smaller variations in the C and N contents. This may suggest a partial saturation of the pores with MTF.

Scanning electron micrograph images were used to analyze the morphologies of the loaded (S1 and S2) and unloaded (KIT-6) silica matrices. Because of the accumulation of KIT-6’s thin threads, the particles in the image appear to be spherical. For the uncharged matrices, the SEM microscopic images showed quasi-spherical particles of different sizes, with small particle sizes and a slight tendency to conglomerate. The charge capacity, distribution, and interaction with MTF molecules may be affected by the aggregation of the KIT-6 mesoporous matrices. A slight degree of aggregation was indicated by the quasi-spherical particles that stuck to each other in the cases of the MTF-loaded matrices (S1 and S2) (Figure 1).

### 3.3. Transmission Electron Microscopy (TEM)

**Figure 2:**
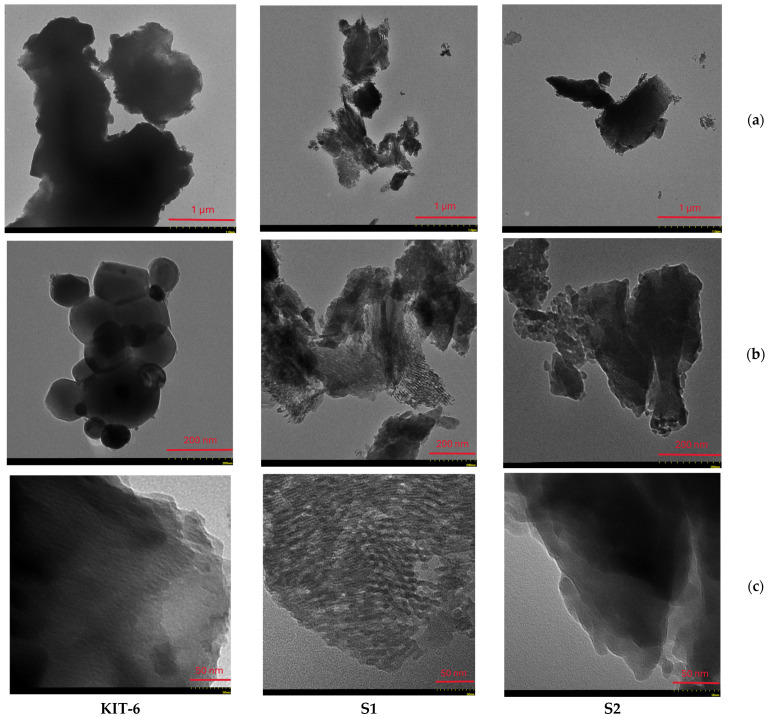
TEM images of KIT-6 sample unloaded, KIT-6 sample S1, and KIT-6 sample S2. The scale bars show (**a**) 1 μm, (**b**) 200 nm, and (**c**) 50 nm.

Transmission electron microscopy images were recorded for three samples of mesoporous silica matrices, denoted as S1, S2 (loaded with MTF), and KIT-6 (the control matrix, unloaded). In Figure 2, clusters of dispersed quasi-spherical matrix particles are highlighted. A porous structure typical of this type of mesoporous material can be observed by the surface’s apparent uniform texture. Figure 2a (1 μm) shows particles of KIT-6 at a lower magnification, showing agglomerations similar to those observed in MCM-48 but with an apparently higher density. At a higher magnification, 200 nm (Figure 2b), the KIT-6 particles appear to be more well defined. The quasi-spherical particle clusters are more obvious and appear to be more compact. The detailed image of the KIT-6 particles—Figure 2c (50 nm)—shows their surface, which appears to be slightly smoother compared to that of MCM-48 particles [22]. This indicates a well-ordered pore structure specific to KIT-6.

### 3.4. Textural Properties: Brunauer–Emmett–Teller (BET)

The registered N_2_ adsorption–desorption isotherms for the unloaded and MTF-loaded silica matrices are shown in Figure S1 (see Supplementary Materials). All the isotherms are of the same shape, which, according to the IUPAC classification, is of type IVa, being characteristic of adsorption in mesoporous adsorbents [24]. This behavior is given by the silica surface–nitrogen molecule interactions and by the interactions between the nitrogen molecules in the condensed state. Each isotherm is accompanied by a clear hysteresis loop, a saturation plateau of a variable length, and an inflection point at higher relative pressures, indicating the presence of microporosity. All the samples exhibit an H1-type hysteresis loop, showing a narrow range of uniform mesopores. Data exploitation from the registered isotherms allowed the determination of the textural parameters characterizing the prepared samples.

Therefore, the surface area available for the adsorption of active molecules is indicated by the specific pore surface area. For the unloaded KIT-6 matrix, the specific surface area has a value of 698.664 m^2^/g; for the charged KIT-6 samples, decreases in the specific surface areas are observed so that for sample S1, we have 143.464 m^2^/g (a 94.05% loading efficiency), and for sample S2, the specific surface area is 88.986 m^2^/g (a 73.16% loading efficiency) (Table 1 and Table 2). As the value approaches close to 1, the correlation coefficient (r^2^) shows how similar it is to the BET experimental model, with very good specific surface area values obtained for S1 (0.9999) and S2 (0.9998) and for the unloaded matrix (KIT-6) (0.9990) (Table 2).

The presence of the micropore volume (which refers to pores smaller than 2 nm) and the micropore area is observed following the KIT-6 matrix, while the disappearance of the microporosity for samples S1 and S2, having values of 0, is found (Table 2). This means that when loading MTF molecules, first, the micropores are occupied, then, the mesopores are filled, explaining the disappearance of the microporosity and the reduced specific surface area and total pore volume.

The P/P0 ratio exhibits a value that is closer to 1, which suggests that the nitrogen was better able to fill all the pores regardless of their size, i.e., it reached the saturation pressure, with the gas molecules being arranged in a multilayered manner. It is observed that with increasing MTF loading, the capillary condensation step moves to higher relative pressures, meaning that the mean pore size increases (see Figure S1 in the Supplementary Materials).

### 3.5. Dissolution Studies

**Figure 3:**
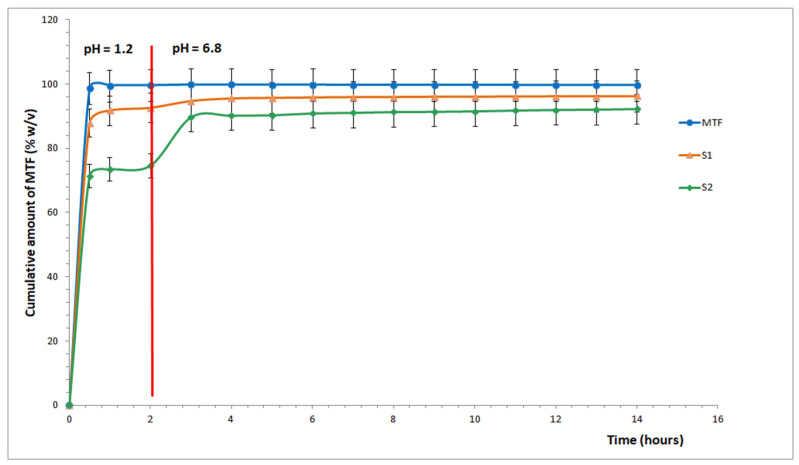
In vitro dissolution release profile of MTF from the mesoporous silica matrix.

Figure 3 displays the experimental results from the in vitro dissolution assay research for KIT-6 samples loaded with MTF at pH 12 at two doses (1 mg/mL and 3 mg/mL).

After the initial sampling at 30 min in the SGF medium (pH 1.2), the percentage of the dosage form that was cleaved ranged from ~71% (S2) to ~88% (S1), reaching a maximum (after 2 h) of ~93% (S1) and ~75% (S2). When an increase in the MTF dissolution capacity is seen for both materials under study, the dissolution medium is changed to SIF (pH 6.8) after 2 h. The proportion of the MTF dissolved at the end of the assay, 12 h later, in the SIF medium varies from ~92% (S2) to ~96% (S1).

When free MTF is utilized as a control with a conventional release, a ~98% total release is seen at the initial sampling, and the recovery is ~100% at the conclusion of the test.

The decision to study the MTF release using a sequential pH change—from pH 1.2 (SGF) to pH 6.8 (SIF) within the same dissolution vessel—was based on the intention to simulate physiological gastrointestinal transit conditions. This sequential pH protocol is widely used to mimic the typical residence time of an oral dosage form in the stomach (~2 h) followed by its transition to the intestinal environment (~10 h), which is especially relevant for modified-release systems.

Our goal was to evaluate how the mesoporous silica matrix responds dynamically to a realistic pH shift over time, as would occur in vivo, rather than isolating the effect of the pH value by keeping the drug in a single medium for the entire duration.

Although the current study design mimics a physiological pH transition, future work could investigate the effects of constant pH conditions (pH 1.2 and 6.8) separately to isolate the influences of the pH value on the release kinetics and matrix behavior.

### 3.6. Analysis of In Vitro Drug Release Kinetics

The correlation coefficient (R^2^), RMSE, and AIC were the criteria used to choose the model that best represents the MTF yield kinetics (Table 3). Regardless of the difference in the concentrations (1 and 3 mg/mL), the Weibull and Korsmeyer–Peppas models provided the best description of the kinetics of the MTF release from the mesoporous KIT-6 matrices, with a correlation coefficient (R^2^) of higher than 0.83 in both situations and the lowest errors. Although both models fit the data well, the Weibull model had slightly better correlation coefficients and AIC values. Table 3 lists the Weibull parameters, where the *a* parameter is the constant of the drug release rate, and parameter *b* indicates the type of release. Both samples displayed values of the *b* parameter lower than 0.75, indicating a diffusion-controlled mechanism. Table 3 presents, also, *n* and *K_P_* values. The Korsmeyer–Peppas model provided valuable mechanistic insights: For both samples, the *n* parameter values were lower than 0.43, confirming the Fickian diffusion behavior.

In addition to RMSE and AIC values, statistical comparisons of the release profiles of formulations S1 and S2 were conducted using both one-way ANOVA and a two-tailed Student’s *t*-tests. The initial release value at time 0 was excluded in subsequent analyses, as it introduced variance unrelated to the release kinetics and could obscure meaningful differences. The one-way ANOVA indicated a significant difference between the samples (F(1,28) = 12.59, *p* = 0.0014). To further assess the significance of the release differences, a Student’s *t*-test was performed. The results showed that S1 exhibited a significantly higher mean release (94.84%) compared to that of S2 (87.61%), with a mean difference of 7.22% (*t* = 5.21, *p* = 0.00013). These findings confirm that the release profile of S1 is significantly greater than that of S2 during the active release phase. All the statistical analyses were performed using a significance level of α = 0.05. The corresponding results are included in the Supplementary Data (Tables S1–S3).

In addition to RMSE and AIC values, statistical comparisons between the release profiles of S1 and S2 were conducted using Student’s *t*-test at each time point (*n* = 3). Significant differences (*p* < 0.05) were observed at specific time points, confirming that the release behaviors differ meaningfully. Furthermore, 95% confidence intervals were calculated for all the model parameters (Table 3), strengthening the reliability of the kinetic-modeling results.

Additionally, we conducted separate modeling of the drug release profiles for the simulated gastric fluid (SGF, pH 1.2) and simulated intestinal fluid (SIF, pH 6.8) phases to gain deeper mechanistic insights. In the SGF, the Korsmeyer–Peppas and Weibull models provided the best fit (R^2^ > 0.997), with Korsmeyer–Peppas parameters indicating non-Fickian diffusion (*n* = 1.1930 for S1; *n* = 1.1836 for S2), likely driven by surface-adsorbed drug or pore-blocking effects contributing to a burst release. The Weibull model parameters (*a* = 0.4244, *b* = 1.2353 for S1; *a* = 0.7715, *b* = 1.2091 for S2) further support this, with *b* > 0.75 indicating a combined diffusion- and erosion-controlled mechanism. For the SIF, the Korsmeyer–Peppas and Weibull models also showed strong fits: *n* < 0.43 for the SIF confirms Fickian diffusion, suggesting a diffusion-dominated release, while *b* < 0.75 indicates a primarily diffusion-controlled mechanism. The pH-dependent mechanisms probably are explained by metformin’s properties: In the acidic SGF, protonation enhances the solubility, promoting rapid release and matrix erosion, while the KIT-6 silica’s surface charge facilitates drug desorption. In the neutral SIF, lower solubility and reduced electrostatic interactions with the negatively charged silica matrix slow the release, favoring diffusion through pores. The plateau phase observed between the third and twelfth hours in both formulations may be attributed to release deceleration due to matrix stabilization or drug–polymer interactions, warranting further investigation into formulation-specific factors. The complete model fitting results, including the kinetic constants and statistical parameters (R^2^, RMSE, and AIC), are available in the Supplementary Materials (Tables S4 and S5).

In comparison with other mesoporous silica materials previously explored for metformin delivery, such as MCM-41, SBA-15, and MCM-48, the KIT-6 matrix exhibited a notably higher drug-loading capacity and a more effective modulation of the release profile. For instance, Sha’at et al. [22] reported that MCM-48-based carriers reached a maximum loading of approximately 94 mg/g and exhibited limited control over the initial burst release. In contrast, the KIT-6 system developed in this study allowed a drug loading of up to 131.69 mg/g. Despite their popularity, MCM-41 and SBA-15 are characterized by linear or hexagonal channel structures that can limit pore accessibility, often resulting in rapid, diffusion-driven releases with minimal modulation [8,9]. KIT-6, with its three-dimensional, bicontinuous cubic architecture, enables a more uniform distribution of the drug within both surface and internal pores, supporting a two-phase release pattern: an initial accelerated release under acidic conditions, followed by prolonged release at a near-neutral pH. Moreover, unlike earlier works, the current study incorporates multifractal analysis to provide a deeper understanding of the release mechanisms, accounting for dynamic structural changes, environmental responsiveness, and memory effects within the matrix. This integrative approach offers valuable insights into the release kinetics of hydrophilic small molecules and supports the development of more predictable, tunable delivery platforms.

## 4. Mathematical Model

We will now consider that a silica-based drug delivery system can be modeled as a fractal or a multifractal mathematical object in both structure and function under certain conditions. In the following, we will outline several reasons supporting the feasibility of this proposition [26,27].

Structural assimilation: Fractals are self-similar entities that are defined by the recurrence of homologous patterns at various scales. Fractals are created through the process of structural assimilation. In the context of drug delivery or controlled release applications, a silica-based drug delivery system commonly contains complex hierarchical structures that may exhibit fractal-like properties. It is possible for silica matrices that are used in medication administration to display branching or porous patterns that display fractal characteristics. Microscopically, the drug release patterns within the complex system may exhibit a self-similar distribution of the drug across various scales. This is dependent on the drug’s ability to diffuse or interact with the silica matrix. Fractal-like patterns can be produced by the drug molecules’ aggregation inside the matrix. The formation of these patterns is contingent upon the drug loading and release mechanisms that are utilized.

Functional assimilation: Fractals possess advantageous characteristics, including self-similarity and scale invariance, that may enhance the functionality of a silica-based drug delivery system. It is possible that these properties will be incorporated into the system and to use a fractal technique in order to define complex drug release patterns. These patterns may include the diffusion of the drug from a silica matrix or the gradual degradation of the medication over time. Due to the scaling qualities that fractals possess, they have the potential to provide insights into the link between the drug release and the dynamics that are reliant on either time or concentration. Moreover, silica-based drug delivery systems frequently exhibit nonlinear release kinetics or intricate behaviors, such as a burst release followed by a controlled release. It is possible to represent these behaviors by employing fractal or multifractal mathematics to encapsulate the complex dynamics of drug interactions with the matrix and the environment in which it is located.

Moreover, fractal geometry may be applied to obtain an understanding of the pore matrix of a drug delivery system. Since these systems usually display scaling correlations between the surface area and mass or diffusion properties, it is possible to use fractals to investigate the surface area and permeability of silica-based drug delivery systems.

The dynamics of silica-based drug delivery systems can be described using the Multifractal Theory of Motion [28], characterized by continuous and nondifferential curves (multifractal curves). This indicates the functionality of the scale covariant derivative

(8) $$\frac{\hat{d}}{dt}=\partial t+{\hat{V}}^{e}\partial_{e}+D^{ie}\partial_{i}\partial_{e},\;\;i,e=1,\;2,\;3$$

where we used the following notations:

(9) $$\left.\begin{array}{c} \partial_{t}=\frac{\partial }{\partial t},\;\;\partial_{e}=\frac{\partial }{\partial x^{e}},\;\;\partial_{i}\partial_{e}=\frac{\partial }{\partial x^{i}}\frac{\partial }{\partial x^{e}}\\ \begin{array}{c} {\hat{V}}^{e}=V_{D}^{e}-iV_{F}^{e},\;\;i=\sqrt{-1}\\ \begin{array}{c} D^{ie}={\frac{1}{4}\left(dt\right)}^{\left[\frac{2}{f\left(\alpha \right)}\right]-1}\left(d^{ie}+i{\bar{d}}^{ie}\right)\\ d^{ie}=\left(\lambda_{+}^{i}\lambda_{+}^{e}-\lambda_{-}^{i}\lambda_{-}^{e}\right),\;\;{\bar{d}}^{ie}=(\lambda_{+}^{i}\lambda_{+}^{e}+\lambda_{-}^{i}\lambda_{-}^{e}) \end{array} \end{array} \end{array}\;\;\;\;\right\}$$

In Equations (8) and (9), the quantities have the meanings given in [29,30].

The explicit form of the tensor $D^{ie}$, i.e., that associated with the multifractal scale transition, is dictated by multifractalization through stochasticity. Therefore, we can distinguish the following types of silica-based drug delivery system dynamics:

(i) Silica-based drug delivery systems’ multifractal dynamics via Markov stochasticity imposed by the following constraints [28,31,32]:
  (10) $$\lambda_{+}^{i}\lambda_{+}^{e}=\lambda_{-}^{i}\lambda_{-}^{e}=-2\lambda \delta^{ie}$$
  where λ is a diffusion-type coefficient associated with the multifractal–nonmultifractal scale transition, and $\delta^{ie}$ is Kronecker’s pseudotensor. For such a system,
  (11) $$D^{ie}\to i\lambda {\left(dt\right)}^{\left[\frac{2}{f\left(\alpha \right)}\right]-1}$$
  so that Equation (8) becomes
  (12) $$\frac{\hat{d}}{dt}=\partial_{t}+{\hat{V}}^{e}\partial_{e}-i\lambda {\left(dt\right)}^{\left[\frac{2}{f\left(\alpha \right)}\right]-1}\partial^{e}\partial_{e}$$
(ii) Silica-based drug delivery systems’ multifractal dynamics by non-Markov stochasticity imposed by the following constraints [28,31]:
  (13) $$d^{ie}=4\alpha^{\delta^{ie}},\;\;{\bar{d}}^{ie}=-4\beta^{\delta^{ie}}$$
  where α and β are the diffusion-type coefficients associated with the multifractal–nonmultifractal scale transition. For such a system,
  (14) $$D^{ie}=\left(\alpha -i\beta \right){\left(dt\right)}^{\left[\frac{2}{f\left(\alpha \right)}\right]-1}$$

Now, Equation (8) becomes

(15) $$\frac{\hat{d}}{dt}=\partial_{t}+{\hat{V}}^{e}\partial_{e}-(\alpha -i\beta )\;{\left(dt\right)}^{\left[\frac{2}{f\left(\alpha \right)}\right]-1}\partial^{e}\partial_{e}$$

Assuming the functionality of the principle of scale covariance, which posits that the laws of physics governing the dynamics of silica-based drug delivery systems remain invariant under spatial, temporal, and scale transformations, various conservation laws can be formulated.

Now, by applying the multifractal operator (12) in the vectorial form to the internal energy per unit of volume (ρε) and adopting the principle of scale covariance [28,31], we obtain the internal energy per unit of volume’s conservation law as follows:

(16) $$\frac{\hat{d}\left(\rho \varepsilon \right)}{dt}=\frac{\partial \left(\rho \varepsilon \right)}{\partial t}+V^{l}\partial_{l}\left(\rho \varepsilon \right)-i\lambda {\left(dt\right)}^{\left[\frac{2}{f\left(\alpha \right)}\right]-1}\partial^{l}\partial_{l}\left(\rho \varepsilon \right)=0$$

In this context, by separating the real part from the imaginary one in Equation (16), we obtain

(17) $$\frac{\partial (\rho \varepsilon )}{\partial t}+\partial_{l}(\rho \varepsilon V_{D}^{l})=(\rho \varepsilon )\partial_{l}V_{D}^{l}$$

(18) $$V_{F}^{l}\partial_{l}(\rho \varepsilon )=-{\lambda^{2}\left(dt\right)}^{\left[\frac{2}{f\left(\alpha \right)}\right]-1}\partial^{l}\partial_{l}\left(\rho \varepsilon \right)$$

It is observable that while there is the transport of internal energy per unit of volume at a differentiable scale, a comparable phenomenon (convection transport) occurs at a fractal scale.

We will now examine the Madelung scenario for modeling the dynamics of silica-based drug delivery systems, as represented by the following differential equations derived from multifractal hydrodynamics [28]:

(19) $$\partial_{t}V_{D}^{i}+\left(V_{D}^{l}\partial_{l}\right)V_{D}^{i}=-\partial^{i}Q$$

(20) $$\partial_{t}\rho +\partial^{i}\left(\rho V_{D}^{i}\right)=0$$

where

(21) $$Q=-2{\lambda^{2}\left(dt\right)}^{\left[\frac{4}{f\left(\alpha \right)}-2\right]}\frac{\partial^{l}\partial_{l}\sqrt{\rho }}{\sqrt{\rho }}=-\frac{V_{F}^{l}V_{Fl}}{2}-{\lambda \left(dt\right)}^{\left[\frac{2}{f\left(\alpha \right)}-1\right]}\partial_{i}V_{F}^{i}$$

(22) $$V_{D}^{i}=2\lambda {\left(dt\right)}^{\left[\frac{4}{f\left(\alpha \right)}-2\right]}\partial^{i}\phi ,\;\;V_{F}^{i}={\lambda \left(dt\right)}^{\left[\frac{2}{f\left(\alpha \right)}-1\right]}\partial^{i}\phi$$

(23) $$\psi =\sqrt{\rho }e^{i\phi },\;\;\bar{\psi }=\sqrt{\rho }e^{-i\phi },\;\;\rho =\psi \bar{\psi }$$

where Q is the specific multifractal potential, ψ is the state function, $\sqrt{\rho }$ is the amplitude, and ϕ is the phase of the state function. We note that the specific multifractal potential may be associated with the following multifractal stress tensor:

(24) $${\hat{\sigma }}_{il}=-2{\lambda^{2}\left(dt\right)}^{\left[\frac{4}{f\left(\alpha \right)}\right]-2}\rho \partial_{i}\partial_{l}ln\rho$$

through the relation

(25) $$\partial^{i}{\hat{\sigma }}_{il}+\rho \partial_{l}Q=0$$

Let us suppose that the silica-based drug delivery system can be regarded as analogous, in both structure and function, to a complex fluid [29].

In such a context, let us reconsider Equations (17), (19) and (20) in planar symmetry (*x*, *y*), with a constraint (22), and $\hat{\sigma }$ in the diagonal form. Also, let us assume that the variation in $\hat{\sigma }$ is induced by the variations in the internal energy per unit of volume and the state density, $\partial_{l}\sigma =\nu \partial_{l}\left(\rho \varepsilon \right)$, with ν=const. Then, in dimensionless variables,

(26) $$\omega t=\tau ,\;\;kx=\xi ,\;\;ky=\eta \;\frac{V_{x}k}{\omega }=V_{\xi },\;\;\frac{V_{y}k}{\omega }=V_{\eta },\;\;\frac{\rho }{\rho_{0}}=N,\;\;\frac{\varepsilon }{\varepsilon_{0}}=\Theta$$

Equations (17), (19), and (20) become

(27) $$\frac{\partial }{\partial \tau }\left(NV_{\xi }\right)+\frac{\partial }{\partial \xi }\left(NV_{\xi }^{2}\right)+\frac{\partial }{\partial \eta }\left(NV_{\xi }V_{\eta }\right)=-\frac{\partial \left(N\Theta \right)}{\partial \xi }\;\frac{\partial }{\partial \tau }\left(NV_{\eta }\right)+\frac{\partial }{\partial \xi }\left(NV_{\xi }V_{\eta }\right)+\frac{\partial }{\partial \eta }\left(NV_{\eta }^{2}\right)=-\frac{\partial \left(N\Theta \right)}{\partial \eta }\;\frac{\partial N}{\partial \tau }+\frac{\partial }{\partial \xi }\left(NV_{\xi }\right)+\frac{\partial }{\partial \eta }\left(NV_{\eta }\right)=0\;\frac{\partial (N\Theta )}{\partial \tau }+\frac{\partial }{\partial \xi }\left(N\Theta V_{\xi }\right)+\frac{\partial }{\partial \eta }\left(N\Theta V_{\eta }\right)=N\Theta \left(\frac{\partial V_{\xi }}{\partial \xi }+\frac{\partial V_{\eta }}{\partial \eta }\right)$$

where the functional scaling relation, $\nu k^{2}/\omega^{2}=1$, is considered. In Equations (20) and (26), $\rho_{0}$ corresponds to the density of the complex fluid at equilibrium, $\varepsilon_{0}$ to the energy of the complex fluid at equilibrium, ω to the complex fluid’s pulsation, and *k* to the inverse of the characteristic length of the complex fluid. For our specific complex fluid, ν will be the square of the acoustic characteristic’s velocity, and σ will be the kinetic pressure.

For numerical integration, we consider the following initial conditions:

(28) $$V_{\xi }\left(0,\xi ,\eta \right)=0,\;\;V_{\eta }\left(0,\xi ,\eta \right)=0\;N\left(0,\xi ,\eta \right)=\frac{1}{3},\;\;\Theta \left(0,\xi ,\eta \right)=\frac{1}{3}\;0\leq \xi \times \eta \leq 1\times 1$$

and the following boundary ones:

(29) $$V_{\xi }\left(\tau ,0,\eta \right)=0,\;\;V_{\xi }\left(\tau ,1,\eta \right)=0,\;V_{\eta }\left(\tau ,0,\eta \right)=0,\;\;V_{\eta }\left(\tau ,1,\eta \right)=0,\;N\left(\tau ,0,\eta \right)=\frac{1}{3},\;\;N\left(\tau ,1,\eta \right)=\frac{1}{3},\;\Theta \left(\tau ,0,\eta \right)=\frac{1}{3},\;\;\Theta \left(\tau ,1,\eta \right)=\frac{1}{3},\;V_{\xi }\left(\tau ,\xi ,0\right)=0,\;\;V_{\xi }\left(\tau ,\xi ,1\right)=0,\;V_{\eta }\left(\tau ,\xi ,0\right)=0,\;\;V_{\eta }\left(\tau ,\xi ,1\right)=0,\;N\left(\tau ,\xi ,0\right)=N_{0}\exp\left[-\frac{{\left(\tau -\frac{1}{3}\right)}^{2}}{(1/3{)}^{2}}\right]\cdot \exp\left[-\frac{{\left(\xi -\frac{1}{2}\right)}^{2}}{(1/3{)}^{2}}\right],\;N\left(\tau ,\xi ,1\right)=\frac{1}{4},\;\;\;\;\;\;\;\;\;\;\;\;\;\;\;\;\;\;\;\;\;\;\;\;\;\;\;\;\;\;\;\;\;\;\;\;\;\;\;\;\;\;\;\;\;\;\;\;\;\;\;\;\;\;\;\;\;\;\;\;\;\;\;\;\;\;\;\;\;\;\;\;\Theta \left(\tau ,\xi ,0\right)=\Theta_{0}\exp\left[-\frac{{\left(\tau -\frac{1}{3}\right)}^{2}}{(1/3{)}^{2}}\right]\cdot \exp\left[-\frac{{\left(\xi -\frac{1}{2}\right)}^{2}}{(1/3{)}^{2}}\right],\;\Theta \left(\tau ,\xi ,1\right)=\frac{1}{3}\;\;\;\;\;\;\;\;\;\;\;\;\;\;\;\;\;\;\;\;\;\;\;\;\;\;\;\;\;\;\;\;\;\;\;\;\;\;\;\;\;\;\;\;\;\;\;\;\;\;\;\;\;\;\;\;\;\;\;\;\;\;\;\;\;\;\;\;\;\;\;$$

In Equation (29), we made the assumption that the perturbation has a spacetime Gaussian profile. Moreover, in the same equations, *N_0_* defines the maximum normalized state density, and $\Theta_{0}$ defines the maximum normalized internal energy per unit of volume.

The equations in (27) were numerically integrated via finite differences [33], based on restrictions (28) and (29).

**Figure 4:**
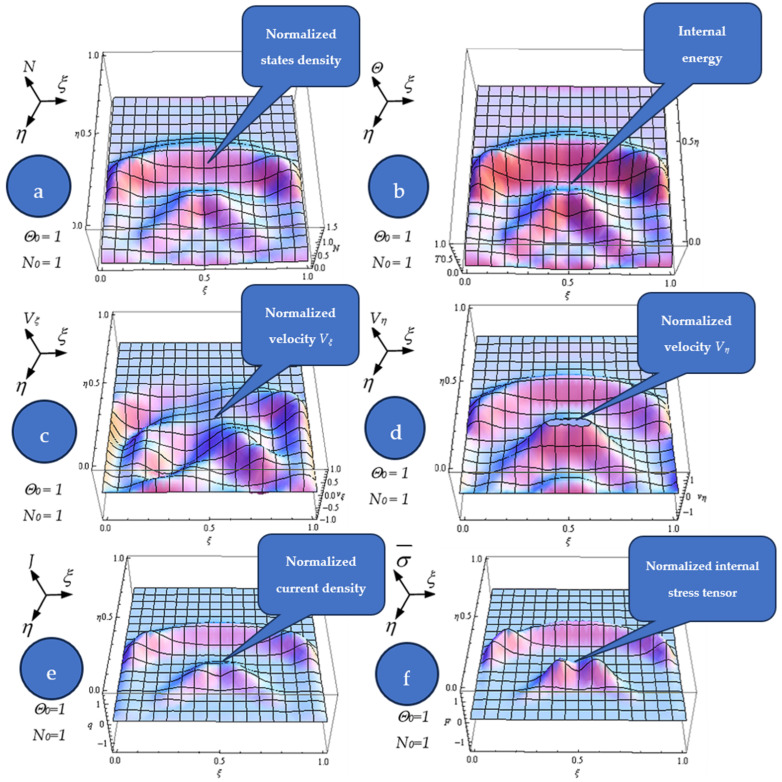
(**a**–**f**) Dependences of the normalized states’ density (*N*) (**a**), internal energy (Θ) (**b**), normalized velocity (*V_ξ_*) (**c**), normalized velocity (*V_η_*) (**d**), normalized current density (*J*) (**e**), and the diagonal component of the normalized internal stress tensor ($\bar{\sigma }$) (**f**) on the normalized spatial coordinates (*ξ*, *η*) at the normalized times *τ* = 0.65 for *N*_0_ = 1 and $\Theta_{0}$ = 1.

In Figure 4a–f, we present the plots for the following:

- The dependences of the normalized states’ density (N);
- The normalized internal energy per unit of volume (Θ);
- The normalized velocity ($V_{\xi }$);
- The normalized velocity ($V_{\eta }$);
- The normalized current density ($J=N{\left(V_{\xi }^{2}+V_{\eta }^{2}\right)}^{1/2}$);
- The diagonal component of the normalized internal stress tensor ($\bar{\sigma }=N\left({V_{\xi }}^{2}+V_{\eta }^{2}\right)$) on the normalized spatial coordinates $\left(\xi ,n\right)$ at the normalized times τ = 0.65 for $N_{0}=1$ and $\Theta_{0}=1$.

**Figure 5:**
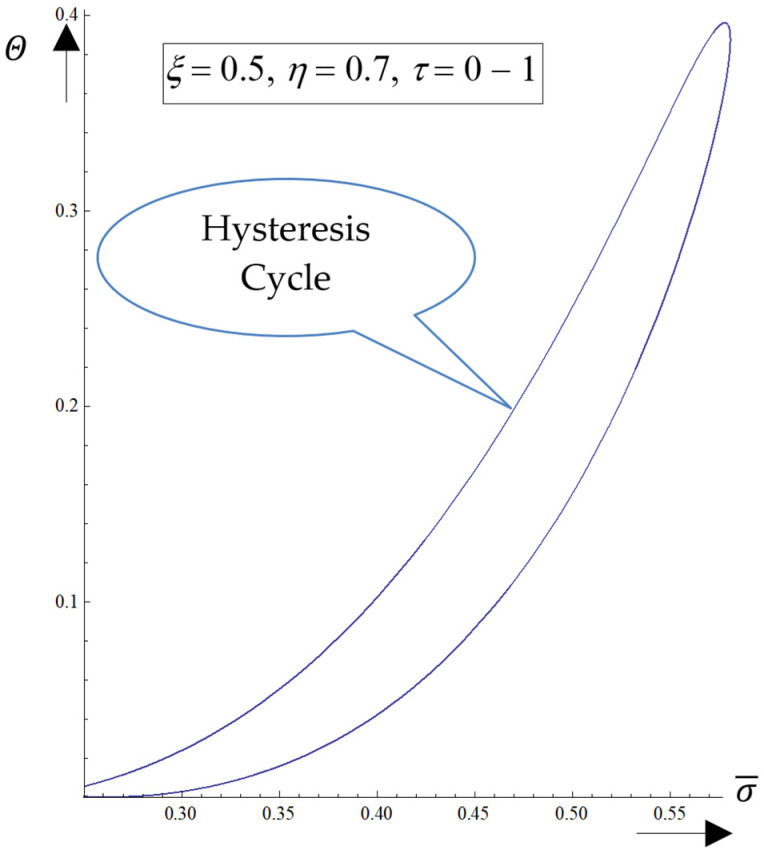
The dependence of the diagonal component of the normalized multifractal stress tensor ($\bar{\sigma }$) on the normalized internal energy (Θ) for *ξ* = 0.5, *η* = 0.7, and *τ* = 0–1 (hysteresis cycle).

The ensuing results are presented:

(i) The generation of structures in a complex fluid through solitons packet solutions [34] (observable as peaks in Figure 4a–f);
(ii) The normalized velocity (*V_ξ_*) (which is normal to the complex fluid streamline) is symmetric with respect to the symmetry axis of the spacetime Gaussian, while vertices are induced at the periphery of the structures of the normalized velocity (*V_η_*) (which is along the “complex fluid streamline”);
(iii) The coupling of the silica-based drug delivery system’s (as a complex fluid) dynamics at a differentiable scale with those at a nondifferentiable scale is achieved through the multifractal stress tensor. Consequently, the drug delivery system entity gains extra kinetic energy (resulting from nondifferentiability), enabling transitions from its own streamline to another;
(iv) By eliminating the temporal gap between the diagonal component of the normalized multifractal stress tensor and the normalized internal energy per unit of volume at various specified points, hysteresis-type effects may be achieved by numerical simulations. For the same *ξ*, such a tendency is more emphasized for low *η* values (see Figure 5);
(v) According to our experimental data, $\bar{\sigma }$ can correspond to the relative pressure (*P/P_0_*), while Θ corresponds to the adsorbed volume (see Figure 5).

Now, if we apply the complex operator (15) to the density of states (ρ) then the conservation law takes the form

(30) $$\frac{\hat{d\rho }}{dt}=\partial_{t}\rho +{\hat{V}}^{e}\partial_{e}\rho -\left(\alpha -i\beta \right){\left(dt\right)}^{\left[\frac{2}{f\left(\alpha \right)}\right]-1}\partial^{e}\partial_{e}\rho =0$$

or better yet, separated at resolution scales

(31) $$\partial_{t}\rho +V_{D}^{e}\partial_{e}\rho +\alpha \;{\left(dt\right)}^{\left[\frac{2}{f\left(\alpha \right)}\right]-1}\partial^{e}\partial_{e}\rho =0$$

at differential scale resolutions, and

(32) $$-V_{F}^{e}\partial \rho -\beta \;{\left(dt\right)}^{\left[\frac{2}{f\left(\alpha \right)}\right]-1}\partial^{e}\partial_{e}\rho =0$$

at nondifferential scale resolutions. Next, if we denote

(33) $$V^{e}=V_{D}^{e}-V_{F}^{e}$$

which is the velocity associated with the multifractal–nonmultifractal scale transition, by adding Equations (31) and (32), the conservation law of the density of states associated with the multifractal–nonmultifractal scale transition is obtained as follows:

(34) $$\partial_{t}\rho +V^{e}\partial_{e}\rho +(\alpha -\beta ){\left(dt\right)}^{\left[\frac{2}{f\left(\alpha \right)}\right]-1}\partial^{e}\partial_{e}\rho =0$$

With respect to Equation (30), let us note the following:

(i) The rates of change of the density field, multifractal convection, and multifractal dissipation of the same field are in equilibrium at any time;
(ii) The presence of the complex dissipative coefficient ($\left(\alpha -i\beta \right){\left(dt\right)}^{\left[\frac{2}{f\left(\alpha \right)}\right]-1}$) specifies that the silica-based drug delivery system exhibits memory, in agreement with works from the literature [35]. In this setting, following each release phase, the silica-based drug delivery system repeats the same cycle at varying resolution scales until the drug concentration diminishes to zero and the silica matrix begins to deteriorate.

We will outline the implications of this differential equation in modeling the dynamics of silica-based drug delivery systems. For this reason, let us examine the differential equation in (20) in the one-dimensional case and subject to the constraint

(35) $$V^{e}\equiv (0,0,V=const.)$$

This becomes

(36) $$\frac{\partial \rho }{\partial t}+V\frac{\partial \rho }{\partial z}-D\frac{\partial^{2}\rho }{\partial z^{2}}=0$$

where we used the following notation:

(37) $$D=(\beta -\alpha )\;{\left(dt\right)}^{\left[\frac{2}{f\left(\alpha \right)}\right]-1}$$

where *D* depends on the physical processes occurring during the drug release from silica matrices and on the scale resolution and its properties.

The model effectively characterizes the nonlinear behaviors of silica-based drug delivery systems across different scale resolutions, adhering to release and diffusion processes during transitions from turbulence to laminar dynamics. From this perspective, the turbulence–laminar transition functions as a multifractal–nonmultifractal scale transition, with *V* representing the drug release rate associated with this transition.

By making the variable change

(38) $$\rho \left(z,t\right)=n\left(z,t\right)exp\;(\frac{Vz}{2D}-\frac{V^{2}t}{4D})$$

which implies

(39) $$\frac{\partial \rho }{\partial t}=(\frac{\partial n}{\partial t}-\frac{V^{2}n}{4D})exp\;(\frac{Vz}{2D}-\frac{V^{2}t}{4D})\;\frac{\partial \rho }{\partial z}=(\frac{\partial n}{\partial z}+\frac{Vn}{2D})exp\;(\frac{Vz}{2D}-\frac{V^{2}t}{4D})\;\frac{\partial^{2}\rho }{\partial z^{2}}=(\frac{\partial^{2}n}{\partial z^{2}}+\frac{V}{D}\frac{\partial n}{\partial z}+\frac{V^{2}n}{4D})exp(\frac{Vz}{2D}-\frac{V^{2}t}{4D})$$

the differential equation in (36) becomes

(40) $$\frac{\partial n}{\partial t}=D\frac{\partial^{2}n}{\partial z^{2}}$$

From the initial and boundary conditions

(41) $$\rho \left(z,0\right)=\rho_{0}=const.\;\rho \left(0,t\right)=0$$

the boundary conditions corresponding to *n* are obtained, i.e.,

(42) $$n\left(z,0\right)=n_{0}exp(\frac{Vz}{2D})\;n\left(0,t\right)=0$$

This problem can be reduced to one without boundary conditions by extending the solution to include the region z<0, where *n* assumes an initial distribution that simulates the boundary condition for z=0 at any time t>0. At the same time, one can choose

(43) $$n\left(z,0\right)=n(-z,0)$$

so that for any extended region, the initial condition will be

(44) $$n\left(z,0\right)=\left\{\begin{array}{c} n_{0}\exp\left(\frac{Vz}{2D}\right),\;z<0\\ -n_{0}\exp\left(-\frac{Vz}{2D}\right),\;z>0 \end{array}\right.$$

The issue at hand is the solution of the diffusion equation for a given initial dimensional distribution within an unbounded medium. This can be addressed by formulating the solution as a Fourier integral as follows:

(45) $$n\left(z,t\right)=\int_{-\infty }^{+\infty }\phi \left(k,t\right)\exp\left(ikz\right)dk$$

(46) $$\phi \left(k,t\right)=\frac{1}{2\pi }\int_{-\infty }^{+\infty }n(z^{\prime },t)\exp\left(-ikz^{\prime }\right)dz^{\prime }$$

After substituting the differential equation in (40), we obtain

(47) $$\frac{\partial \phi }{\partial t}+k^{2}D\phi =0$$

with the solution

(48) $$\phi \left(k,t\right)=\phi \left(k,0\right)exp(-k^{2}Dt)$$

where $\phi \left(k,0\right)$ was obtained by substituting the initial distribution (*n*) into Equation (46). Therefore, Equation (45) can be expressed as an integral over the entire initial distribution as follows:

(49) $$n\left(z,t\right)=\frac{1}{2\pi }\int_{-\infty }^{+\infty }\int_{-\infty }^{+\infty }n(z^{\prime },0)\exp\left(-k^{2}Dt\right)exp\left[ik(z-z^{\prime })\right]dz^{\prime }dk$$

Once the integral above is solved with respect to *k*, solving it for *n* becomes straightforward, leading to

(50) $$n\left(z,t\right)=\frac{1}{{\left(4\pi Dt\right)}^{\frac{1}{2}}}\int_{-\infty }^{+\infty }n(z^{\prime },0)\exp\left[-\frac{{\left(z-z^{\prime }\right)}^{2}}{4Dt}\right]dz^{\prime }$$

The solution can be completed by substituting Equation (44) into Equation (50) and calculating the integral. The result for $\rho \left(z,t\right)$ is given by the following equation:

(51) $$\rho \left(z,t\right)=\frac{n_{0}}{2}\exp\left(-\frac{Vt}{D}\right)+\frac{n_{0}}{2}\exp\left(-\frac{Vt}{D}\right)\cdot \left[\frac{erf(z-Vt)}{{\left(4Dt\right)}^{\frac{1}{2}}}+\frac{erf(z+Vt)}{{\left(4Dt\right)}^{\frac{1}{2}}}\right]$$

where $\mathrm{erf}\left(x\right)$ is the Laplace function given by

(52) $$\mathrm{erf}\left(x\right)=\frac{1}{2\sqrt{\pi }}\int_{0}^{\infty }\exp\left(-x^{2}\right)dx$$

with the values of this function being tabulated.

Now, by a convenient choice of variables, in dimensionless coordinates, Equation (51) can be written as follows:

(53) $$\rho \left(x,y\right)=\exp\left(-x\right)+exp(-x)\left\{erf\left[\frac{\left(y-x\right)}{\sqrt{x}}\right]+erf\left[\frac{\left(y+x\right)}{\sqrt{x}}\right]\right\}$$

**Figure 6:**
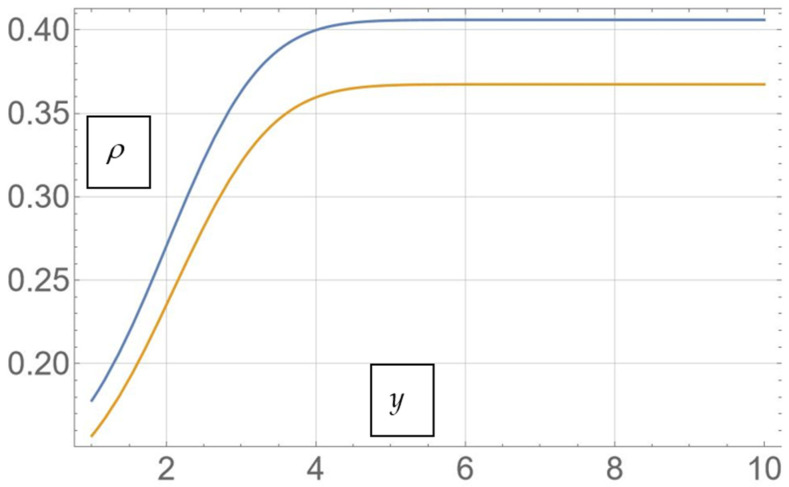
Theoretical drug release curves for two distinct resolution scales: ρ(2,y)—orange—to ρ(2.1,y)—blue.

We present, in Figure 6, the theoretical in vitro dissolution release profile of the MTF from the mesoporous silica matrix for two distinct resolution scales: ρ(2,y)—orange curve—and ρ(2.1,y)—blue curve. The resolution scales can be assigned to samples S1 and S2 from the experimental data.

**Figure 7:**
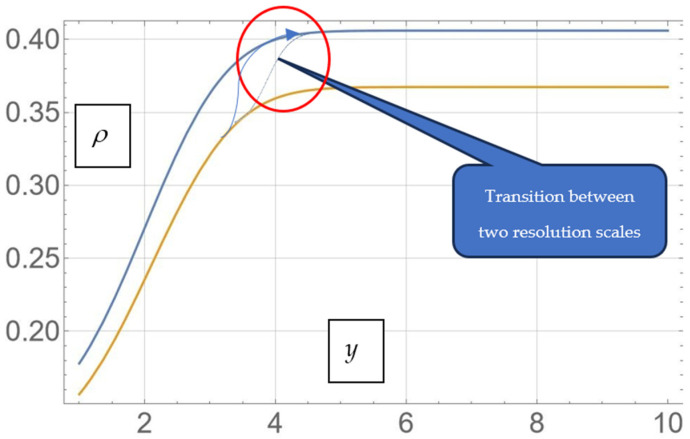
Theoretical drug release curves corresponding to the transition between two distinct resolution scales from ρ(2,y)—orange—and ρ(2.1,y)—blue.

In Figure 7, we present the correspondence between the same two release curves realized by the transition between two resolution scales. The system evolves on the first curve up to the saturation level, corresponding to the first resolution scale (and, therefore, to a morphology), after which, the system starts the same process again at another resolution scale (and, therefore, at another morphology).

## 5. Conclusions

This study successfully demonstrated the development of an innovative drug delivery system based on KIT-6 mesoporous silica matrices loaded with metformin hydrochloride. The synthesized matrices exhibited a large surface area and a tunable pore size, favoring efficient drug encapsulation. The experimental results showed that the degree of drug loading increased with higher initial drug concentrations, while SEM, TEM, and BET analyses confirmed that the structural integrity and morphological characteristics were consistent with the controlled release functionality. In vitro dissolution studies revealed that the drug release occurred in two stages: an initial rapid release under acidic conditions, followed by a slower and sustained release in simulated intestinal fluid. Kinetic modeling identified the Weibull and Korsmeyer–Peppas models as the most appropriate, suggesting a diffusion-controlled mechanism. In conclusion, the kinetic modeling results indicate that metformin release from the KIT-6 mesoporous silica matrix follows a biphasic, predominantly diffusion-controlled mechanism. The best fits were obtained with the Weibull and Korsmeyer–Peppas models, with *b* below 0.75 and *n* values below 0.43, confirming Fickian diffusion behavior. The rapid initial release in SGF corresponds to the surface-adsorbed or near-surface drug, while the slower release in SIF suggests diffusion through the porous network. These findings demonstrate that the release mechanism is governed primarily by matrix-controlled diffusion, with significant contributions from the physicochemical environment and pore structure.

We have shown that the dynamics of the drug release from mesoporous silica matrices can be accurately described by multifractal theory. Utilizing multifractal curves and the scale covariant derivative allows for capturing complex and nonlinear drug release processes.

The multifractal analysis revealed dual-scale energy transport phenomena: conventional transport at the differentiable scale and convection-like transport occurring at the fractal scale.

Numerical simulations indicate the formation of soliton packet solutions, reflecting structured release patterns within the complex fluid analogy of silica matrices. Distinct behaviors for velocities normal and parallel to streamlines further clarify how fractal dynamics govern transport mechanisms.

The mathematical modeling demonstrates that drug release occurs through transitions between resolution scales, each associated with distinct morphologies of the mesoporous matrix. These scale transitions explain the experimental observations of hysteresis phenomena and complex kinetic profiles, underscoring the memory effects within silica-based drug delivery systems.

Overall, the study validates the utility of KIT-6 as a robust carrier for oral antidiabetic therapy and lays the groundwork for future investigations into similar systems targeting other therapeutic molecules.

## Figures and Tables

**Table 1:** Experimental data on the degrees of loading of the mesoporous silica matrices.

Sample	Concentration Sol. (mg/mL)	pH	Metformin Load Grade (mg/g)	Metformin-Loading Yields (wt.%)
S1	1	12	56.432 ± 0.1339 (SD)	94.05
S2	3		131.686 ± 0.1261 (SD)	73.16

SD: standard deviation (*n* = 3).

**Table 2:** Textural properties of the prepared mesoporous silica matrices.

Sample Code	Specific Surface Area S_BET_ (m^2^/g)	Correlation Coefficient with BET Model (r^2^)	External Surface Area (m^2^/g)	Total Pore Volume (cc/g)	Micropore Volume (cm^3^/g)	Micropore Area (m^2^/g)
KIT-6	698.664 ± 0.2741	0.9990	441.051 ± 0.2094	0.619	0.131	257.613
S1	143.464 ± 0.0765	0.9999	143.464 ± 0.0765	0.268	0	0
S2	88.986 ± 0.0942	0.9998	88.986 ± 0.0942	0.200	0	0

S_BET_: Brunauer–Emmett–Teller surface area analysis; SD: standard deviation (*n* = 3).

**Table 3:** Parameter values of the kinetic release.

Kinetic Model	Model Coefficients	Mesoporous Silica (KIT-6)	
		S1	S2
Zero-order	*K*_0_	0.3931 (0.1777, 0.6085)	1.2852 (0.614, 1.957)
	R^2^	0.5437	0.5696
	RMSE	1.5372	4.7688
	AIC	57.5194	91.4836
First-order	*K*_1_	0.0042 (0.0018, 0.0066)	0.0156 (0.0072, 0.0240)
	R^2^	0.5359	0.5601
	RMSE	1.5482	4.9156
	AIC	57.7340	92.3930
Higuchi	*K_H_*	41.8991 (25.6767, 68.2984)	38.5740 (24.0163, 61.9738)
	R^2^	0.2762	0.3692
	RMSE	37.8781	30.7756
	AIC	153.6519	147.4224
Korsmeyer–Peppas	*n*	0.0242 (0.0188, 0.0296)	0.0852 (0.0623, 0.1081)
	*K_P_*	91.1381 (90.3290, 91.9489)	75.9451 (72.8366, 79.2057)
	R^2^	0.8741	0.8333
	RMSE	0.8129	3.0724
	AIC	38.4087	78.2936
Weibull	*a*	0.4077 (0.4021, 0.4277)	0.6886 (0.6369, 0.7708)
	*b*	0.1254 (0.1029, 0.1479)	0.2411 (0.1807, 0.3015)
	R^2^	0.9187	0.8523
	RMSE	0.6075	2.7182
	AIC	29.6664	74.6196
Hixson–Cromwell	*K_HC_*	0.0064 (0.0039, 0.0089)	0.0226 (0.0105, 0.0347)
	R^2^	0.5385	0.5632
	RMSE	1.5443	4.8605
	AIC	57.6594	92.0546
Baker–Lonsdale	*K_BL_*	0.0059 (0.0029, 0.0089)	0.0110 (0.0058, 0.0162)
	R^2^	0.6015	0.6164
	RMSE	94.8646	87.9157
	AIC	181.1943	178.9121

## Data Availability

All the data are presented in the manuscript.

## Supplementary Materials

The following supporting information can be downloaded at https://www.mdpi.com/article/10.3390/pharmaceutics17070882/s1: Figure S1: (a) Nitrogen adsorption–desorption isotherms of KIT-6 sample unloaded, S1, and S2; pore size distributions obtained from (b) adsorption and (c) desorption curves; Table S1: ANOVA (calculated by including the initial release value at time 0); Table S2: ANOVA (calculated by excluding the initial release value at time 0); Table S3: T-test results comparing drug release profiles of metformin-hydrochloride-loaded KIT-6 mesoporous silica matrices (S1 and S2 formulations); Table S4: Fitting parameters and statistical evaluation of MTF release from KIT-6 matrices in simulated gastric fluid (pH 1.2) using various kinetic models; Table S5: Fitting parameters and statistical evaluation of MTF release from KIT-6 matrices in simulated intestinal fluid (pH 6.8) using various kinetic models.

## Abbreviations

AIC: Akaike information criterion; BET: Brunauer-Emmett-Teller; DDS: Drug delivery systems; HPLC: High Performance Liquid Chromatography; MSNs: Mesoporous silica nanoparticles; MTF: Metformin hydrochloride; RMSE: Root mean square error; SD: Standard deviation; SEM: Scanning Electron Microscopy; SGF: Simulated gastric fluid; SIF: Simulated intestinal fluid; TEM: Transmission Electron Microscopy; TEOS: Tetraethyl orthosilicate.
