# Supplementary material for: Development of Modified Drug Delivery Systems with Metformin Loaded in Mesoporous Silica Matrices: Experimental and Theoretical Designs
Source: Pharmaceutics. 2025 Jul 4;17(7):882. doi: 10.3390/pharmaceutics17070882 (PMC12298488; doi:10.3390/pharmaceutics17070882)
Supplement: Supplementary file 1 [file pharmaceutics-17-00882-s001.zip › pharmaceutics-3674553-supplementary.pdf]

3.4. Textural Properties: Brunauer-Emmett-Teller (BET)

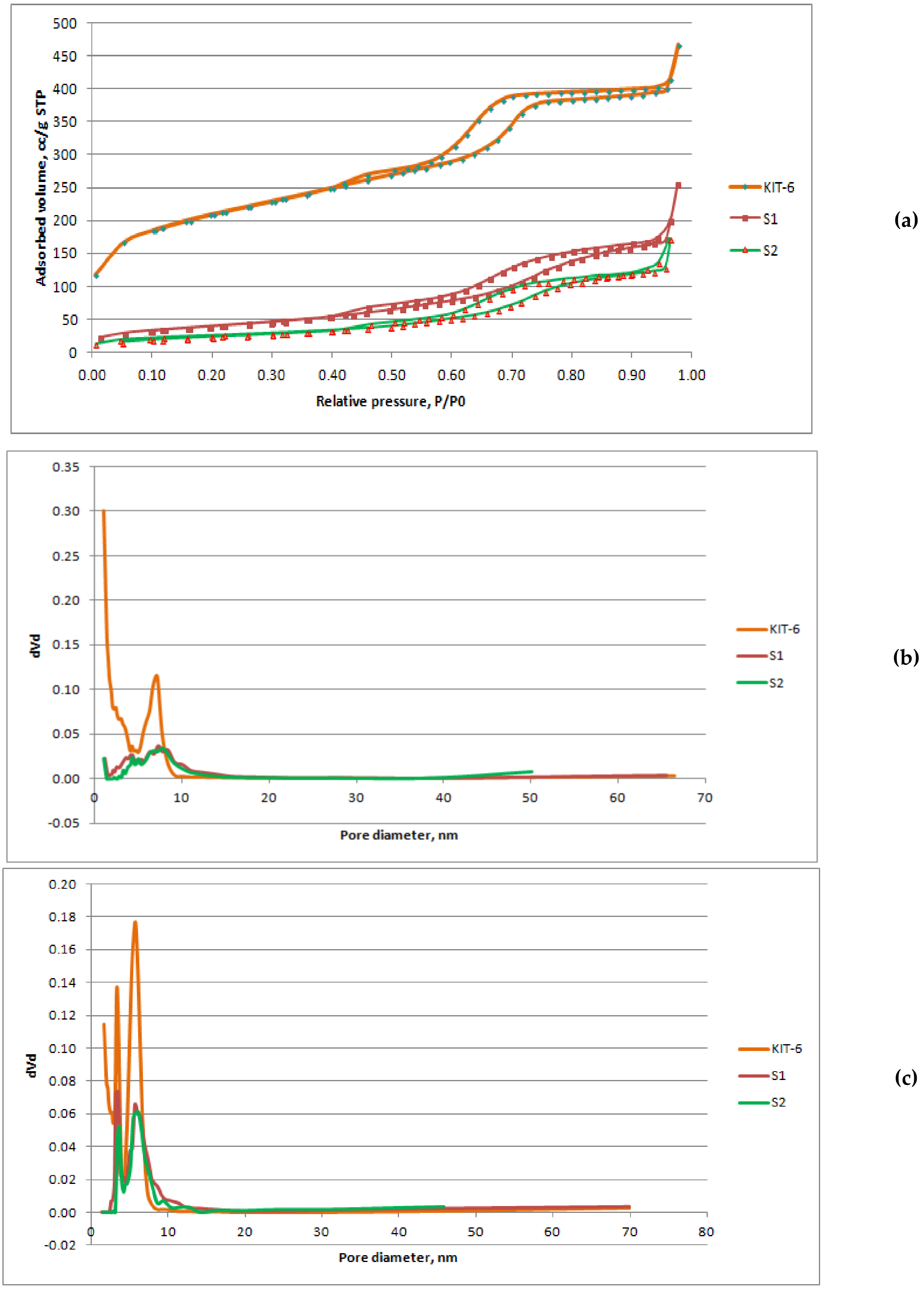

Figure S1. (a) Nitrogen adsorption-desorption isotherms of KIT-6 sample unloaded, S1, and S2; pore size distributions obtained from **(b)** adsorption and **(c)** desorbtion curves.

3.6. Analysis of In Vitro Drug Release Kinetics

Table S1. ANOVA (calculation made including initial release value at time 0).

Source of Variation	SS	df	MS	F	*p*-Value	F critical
Between Groups	366.7986	1	366.7986	0.6669	0.4205	4.1708
Within Groups	16498.6375	30	549.9545			
Total	16865.4361	31				
Alpha - 0.05						

Table S2. ANOVA (calculation made excluding initial release value at time 0).

Source of Variation	SS	df	MS	F	*p*-Value	F critical
Between Groups	391.2518	1	391.2518	12.5906	0.0013	4.1959
Within Groups	870.0910	28	31.0746			
Total	1261.3429	29				
Alpha - 0.05						

Table S3. T-test results comparing drug release profiles of metformin hydrochloride-loaded KIT-6 mesoporous silica matrices (S1 and S2 formulations).

**Parameter Value**	
**Mean (S1)**	94.8373
**Mean (S2)**	87.6147
**Variance (S1)**	5.5483
**Variance (S2)**	56.6010
**Observed Mean Difference**	7.2227
**Variance of the Differences**	28.8223
p	0.000132
**t Statistic**	5.2105
**t Critical two-tail**	2.1448

Table S4. Fitting Parameters and Statistical Evaluation of MTF Release from KIT-6 Matrices in Simulated Gastric Fluid (pH = 1.2) Using Various Kinetic Models

Model	Set	Parameters	R^2	RMSE	AIC
**Zero-order**	S1	K_0 = 37.7943	0.5050	27.6692	36.1077
	S2	K_0 = 30.3440	0.5006	22.4110	34.4216
**First-order**	S1	K_1 = 11.0300	0.4681	11337.4276	84.2321
	S2	K_1 = 10.9421	0.4679	8740.4400	82.1509
**Higuchi**	S1	K_H = 1.6528	0.8110	77.1009	44.3061
	S2	K_H = 1.4070	0.8430	62.1247	42.5783
**Korsmeyer-Peppas**	S1	K_P = 89.2639<br>n = 1.1930	0.9977	60.8700	42.4151
	S2	K_P = 72.0179<br>n = 1.1836	0.9977	48.7111	40.6324
**Weibull**	S1	a = 0.4244<br>b = 1.2353	0.9981	12.8222	29.9546
	S2	a = 0.7715<br>b = 1.2091	0.9978	17.5331	32.4579
**Hixson-Crowell**	S1	K_{HC} = 1.8220	0.4796	52.9104	41.2939
	S2	K_{HC} = 1.6933	0.4780	42.6851	39.5759
**Baker-Lonsdale**	S1	K_{BL} = 0.1327	0.6059	78.5974	44.4599
	S2	K_{BL} = 0.0637	0.5610	63.3976	42.7406

Table S5. Fitting Parameters and Statistical Evaluation of MTF Release from KIT-6 Matrices in Simulated Intestinal Fluid (pH = 1.2) Using Various Kinetic Models

**Model**	**Set**	**Parameters**	**R^2**	**RMSE**	**AIC**
**Zero-order**	S1	K_0 = 0.0969	0.6936	0.2222	2.2778
	S2	K_0 = 0.2206	0.9559	0.1636	9.6304
**First-order**	S1	K_1 = 0.0010	0.6914	0.2226	2.2339
	S2	K_1 = 0.0024	0.9544	0.1659	9.2856
**Higuchi**	S1	K_H = 34.5415	0.7707	21.2609	107.1837
	S2	K_H = 32.8680	0.9829	19.7958	105.4702
**Korsmeyer-Peppas**	S1	K_P = 94.2611<br>n = 0.0083	0.8426	0.1589	10.3148
	S2	K_P = 87.8879<br>n = 0.0182	0.9901	0.0774	27.5907
**Weibull**	S1	a = 0.0576<br>b = 0.3527	0.8689	0.1502	11.6864
	S2	a = 0.0767<br>b = 0.4803	0.9886	0.0838	25.6962
**Hixson-Crowell**	S1	K_{HC} = 0.0015	0.6922	0.2225	2.2486
	S2	K_{HC} = 0.0036	0.9549	0.1652	9.4004
**Baker-Lonsdale**	S1	K_{BL} = 0.0018	0.7170	95.8642	143.3293
	S2	K_{BL} = 0.0027	0.9646	91.2233	142.1383
